# Entropy Analysis in Double-Diffusive Convection in Nanofluids through Electro-Osmotically Induced Peristaltic Microchannel

**DOI:** 10.3390/e21100986

**Published:** 2019-10-10

**Authors:** Saima Noreen, Sadia Waheed, Abid Hussanan, Dianchen Lu

**Affiliations:** 1 Department of Mathematics, Faculty of Science, Jiangsu University, Zhenjiang 212013, China; laurel_lichen@yahoo.com (S.N.); dclu@ujs.edu.cn (D.L.); 2 Department of Mathematics, COMSATS University Islamabad 45550, Tarlai Kalan Park Road, Islamabad 44000, Pakistan; sadiawaheed2017@gmail.com; 3 Division of Computational Mathematics and Engineering, Institute for Computational Science, Ton Duc Thang University, Ho Chi Minh City 700000, Vietnam; 4 Faculty of Mathematics and Statistics, Ton Duc Thang University, Ho Chi Minh City 700000, Vietnam

**Keywords:** entropy generation, double-diffusive convection, electro-osmosis, nanofluid, bio-microfluidics, exact solution

## Abstract

A theoretical study is presented to examine entropy generation in double-diffusive convection in an Electro-osmotic flow (EOF) of nanofluids via a peristaltic microchannel. Buoyancy effects due to change in temperature, solute concentration and nanoparticle volume fraction are also considered. This study was performed under lubrication and Debye-Hückel linearization approximation. The governing equations are solved exactly. The effect of dominant hydrodynamic parameters (thermophoresis, Brownian motion, Soret and Dufour), Grashof numbers (thermal, concentration and nanoparticle) and electro-osmotic parameters on double-diffusive convective flow are discussed. Moreover, trapping, pumping, entropy generation number, Bejan number and heat transfer rate were also examined under the influence of pertinent parameters such as the thermophoresis parameter, the Brownian motion parameter, the Soret parameter, the Dufour parameter, the thermal Grashof number, the solutal Grashof number, the nanoparticle Grashof number, the electro-osmotic parameter and Helmholtz–Smoluchowski velocity. The electro-osmotic parameter powerfully affected the velocity profile. The magnitude of total entropy generation increased as the thermophoresis parameter and Brownian motion parameter increased. Soret and the Dufour parameter had a strong tendency to control the temperature profile and Bejan number. The findings of the present analysis can be used in clinical purposes such as cell therapy, drug delivery systems, pharmaco-dynamic pumps and particles filtration.

## 1. Introduction

In 1809, Resuss [1] illustrated an electrokinetic phenomenon known as electroosmosis or Electro-osmotic flow (EOF). It is defined as the flow of fluid in any conduit (e.g., microchannel, capillary tube) under the effect of the external electric field. EOF has many applications in micro-flow injection analysis, micro-liquid chromatography and micro-energy system. Patankar et al. [2] investigated the behavior of electro-osmotic flow through a microchannel. Yang et al. [3] studied the EOF in microfluidics. Tang et al. [4] scrutinized the EOF for a Power-law model. Later, Miller et al. [5] predicted EOF through a carbon nanotube membrane.

Peristaltic transport of a fluid is a special type of fluid transport that occurs due to the propagation of progressive waves of area contraction/expansion. This is a natural process of transport in living beings. The literature reveals that different studies have explained this phenomenon under various impacts. Some recent readings are cited in [6,7,8,9,10,11].

Nanofluids have been found to exhibit enhanced thermophysical properties (thermal conductivity, thermal diffusivity, the viscosity of convective heat transport coefficient) compared to base fluids. Nanofluids dynamics have shown tremendous potential applications in many fields. The endoscopic impact of nanofluid in peristalsis was analyzed by Akbar et al. [12]. Noreen [13] investigated nanofluids under induced magnetic field and mixed convection. Furthermore, Tripathi et al. [14] studied the peristaltic motion of nanofluid. Reddy et al. [15] recommended the peristaltic flow of nanofluid through complaint walls. Ebaid et al. [16] examined the peristaltic motion of nanofluid with convective conditions.

Shao et al. [17] studied a reference solution for double-diffusion convection. Double-diffusive convection is a fluid dynamics phenomenon that describes a form of convection driven by two different density gradients (temperature and concentration) that have different diffusion rates. If the temperature difference is held constant, thermal diffusion produces a concentration gradient. Considering the wide applications of double-diffusive convection, it was studied in a nanofluidic flow model with peristaltic pumping. Huppert et al. [18] discussed the applications of double-diffusion convection. Similarly, Bég et al. [19] described the peristaltic pumping of nanofluids through double-diffusive convection. Kefayati [20] explained double-diffusion convection for pseudoplastic fluids. The peristaltic motion of MHD flow through double-diffusive convection was studied by Rout et al. [21]. Gaffar et al. [22] observed the double-diffusive convection under the effect of heat absorption. Furthermore, Mohan et al. [23] also described the influence of double-diffusive convection in a lid-driven cavity.

Peristaltic flow and EOF have guaranteed biomedical and engineering applications. Today, the collective effect of peristalsis and electroosmosis is of supreme importance in many applications, such as biomicrofluidic devices and biomimetic pumping. Some mathematical models with various physical applications and various flow regimes have been studied. Bandopadhyay et al. [24] inspected peristaltic motion of viscous fluid in the microchannel. Unsteady electrokinetic transport through peristaltic microchannel was studied by Tripathi et al. [25]. Tripathi et al. [26] explored the peristaltic motion of the couple-stress fluid through the microchannel. Prakash et al. [27] examined the peristaltic motion of Williamson ionic nanofluid in the tapered microchannel. Tripathi et al. [28] also studied blood flow modulated by electroosmosis. Moreover, Tripathi et al. [29] worked on the combined effect of electroosmosis and peristalsis through the microchannel. In addition, Tripathi et al. [30] revealed the peristaltic transport of nanofluids with buoyancy effect via a microchannel. The impact of heat and mass transport for the combined effect of electroosmosis and peristalsis was studied by Waheed et al. [31]. Noreen et al. [32] scrutinized the effect of heat on EOF through peristaltic pumping. In another study, Noreen et al. [33] recommended the transport of MHD nanofluid through the peristaltic microchannel. 

Entropy generation in peristaltically induced microchannels is a rapidly emerging area of interest. Entropy production is related to thermodynamic irreversibility, which exists in all types of heat and mass transfer processes. The study of entropy generation within the system is important because it helps to track the sources of the available energy. Different sources of irreversibility include heat flow through the finite temperature gradient, convective heat transfer characteristics, viscosity and diffusion effects. Entropy can be minimized to preserve the energy quality for the optimal design of any thermal system. At present, the research topic of minimizing entropy generation has gained special status amongst scientists worldwide. Limited studies have investigated the entropy production in electro-osmotically induced peristaltic microchannels. Kefayati [34] explained entropy production for Non-Newtonian nanofluids through a porous cavity. Kefayati [35] has also described entropy production and double-diffusive convection for power-law fluids. In his study [36], the entropy production in double-diffusive convection in an open cavity was explained. He also explained entropy production for EOF through FDLBM simulation [37]. Ranjit et al. [38] explained entropy production for EOF regulated by peristaltic pumping. Bhatti et al. [39] analyzed entropy production for nanofluid through a microchannel. Kefayati [40] presented the simulation of entropy production using Buongiorno’s mathematical model. Kefayati et al. [41,42] discovered entropy production for MHD flow and inclined channel flow. Ranjit et al. [43] demonstrated an entropy analysis for EMHD flow through an asymmetric microchannel. Moreover, Noreen et al. [44] studied the entropy production in peristaltically flowing viscous fluid. 

All the above-cited work was obtained through two solutions approaches, i.e., analytical and numerical. The authors computed the exact solutions, as they are error free and more efficient than numerical solutions. This study was motivated by applications in novel nanofluid drug delivery systems where a small volume of drugs can be transported in the diseased portion of physiological vessels with the help of organ-on-chip devices. Thermal enhancement mechanism analysis of double-diffusive convection in nanofluids transported by peristaltic microchannel has not been discussed previously. Therefore, the current investigation aimed to fill this gap. The findings of the present analysis can be used in clinical purposes such as cell therapy, drug delivery systems, pharmaco-dynamic pumps and particles filtration. In addition, in order to deepen our understanding of thermosolutal convection, special attention was paid to study entropy generation. The detailed mathematical formulation is given and solution expressions are also reported.

## 2. Mathematical Formulation

### 2.1. Flow Regime

**Figure 1:**
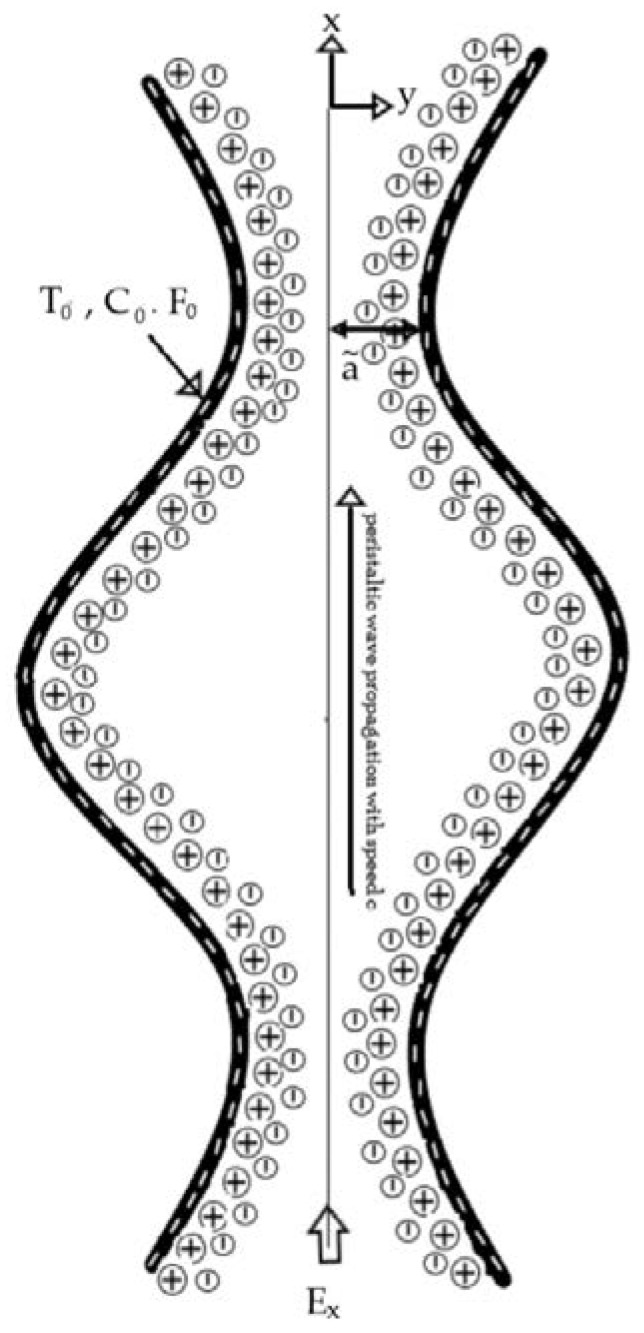
Schematic of the geometry.

The physical model (shown in Figure 1) consists of a two-dimensional EOF of nanofluid with double-diffusive convection through a microfluidic channel. 

The medium is induced by propagating a peristaltic wave at velocity c along the microchannel. The temperature, solutal concentration and nanoparticle fraction for the lower channel wall are taken as $T_{0},C_{0}$ and $F_{0}$, respectively.

Mathematically, microchannel wall’s geometry is expressed as [23]

(1) $$\tilde{h}\left(\tilde{X},\tilde{t}\right)=\tilde{a}+\tilde{b}\sin\left(2\pi \frac{\left(\tilde{X}-c\tilde{t}\right)}{\lambda }\right)$$

where $\tilde{h}$ represents the transverse vibration of the wall, $\tilde{a}$ is the half width of channel, $\tilde{b}$ is the amplitude of wave, $\tilde{X}$ represents the axial coordinate, c is the speed of the wave, λ represents the wavelength and $\tilde{t}$ is the time.

### 2.2. Governing Equations

The equations governing the physical flow problem for nanofluid with double-diffusive convection in electrohydrodynamic (EHD) are [19]

(2) $$\frac{\partial \tilde{U}}{\partial \tilde{X}}+\frac{\partial \tilde{V}}{\partial \tilde{Y}}=0.$$

(3) $$\rho_{f}\left(\frac{\partial \tilde{U}}{\partial \tilde{t}}+\tilde{U}\frac{\partial \tilde{U}}{\partial \tilde{X}}+\tilde{V}\frac{\partial \tilde{U}}{\partial \tilde{Y}}\right)=-\frac{\partial \tilde{P}}{\partial \tilde{X}}+\mu \left(\frac{\partial^{2}\tilde{U}}{\partial {\tilde{X}}^{2}}+\frac{\partial^{2}\tilde{U}}{\partial {\tilde{Y}}^{2}}\right)+g\{\left(1-F_{0}\right)\rho_{0}\left(\beta_{t}\left(\tilde{T}-T_{0}\right)+\;\beta_{c}\left(\tilde{C}-C_{0}\right)\right)-\left(\rho_{p}-\rho_{0}\right)\left(\tilde{F}-F_{0}\right)\}+\rho_{e}E_{x},$$

(4) $$\rho_{f}\left(\frac{\partial \tilde{V}}{\partial \tilde{t}}+\tilde{U}\frac{\partial \tilde{V}}{\partial \tilde{X}}+\tilde{V}\frac{\partial \tilde{V}}{\partial \tilde{Y}}\right)=-\frac{\partial \tilde{P}}{\partial \tilde{Y}}+\mu \left(\frac{\partial^{2}\tilde{V}}{\partial {\tilde{X}}^{2}}+\frac{\partial^{2}\tilde{V}}{\partial {\tilde{Y}}^{2}}\right)+g\left\{\left(1-F_{0}\right)\rho_{0}\left(\beta_{t}\left(\tilde{T}-T_{0}\right)+\;\beta_{c}\left(\tilde{C}-C_{0}\right)\right)-\left(\rho_{p}-\rho_{0}\right)\left(\tilde{F}-F_{0}\right)\right\}.$$

(5) $${\left(\rho C_{p}\right)}_{f}\left(\frac{\partial \tilde{T}}{\partial \tilde{t}}+\tilde{U}\frac{\partial \tilde{T}}{\partial \tilde{X}}+\tilde{V}\frac{\partial \tilde{T}}{\partial \tilde{Y}}\right)=k\left(\frac{\partial^{2}\tilde{T}}{\partial {\tilde{X}}^{2}}+\frac{\partial^{2}\tilde{T}}{\partial {\tilde{Y}}^{2}}\right)+D_{tc}\left(\frac{\partial^{2}\tilde{C}}{\partial {\tilde{X}}^{2}}+\frac{\partial^{2}\tilde{C}}{\partial {\tilde{Y}}^{2}}\right)+{\left(\rho C_{p}\right)}_{p}\{D_{b}(\frac{\partial \tilde{F}\partial \tilde{T}}{\partial \tilde{X}\partial \tilde{X}}+\frac{\partial \tilde{F}\partial \tilde{T}}{\partial \tilde{Y}\partial \tilde{Y}})+\frac{D_{t}}{T_{0}}(({\frac{\partial \tilde{T}}{\partial \tilde{X}})}^{2}+{\left(\frac{\partial \tilde{T}}{\partial \tilde{Y}}\right)}^{2})\}.$$

(6) $$\left(\frac{\partial \tilde{C}}{\partial \tilde{t}}+\tilde{U}\frac{\partial \tilde{C}}{\partial \tilde{X}}+\tilde{V}\frac{\partial \tilde{C}}{\partial \tilde{Y}}\right)=D_{s}\left(\frac{\partial^{2}\tilde{C}}{\partial {\tilde{X}}^{2}}+\frac{\partial^{2}\tilde{C}}{\partial {\tilde{Y}}^{2}}\right)+D_{ct}\left(\frac{\partial^{2}\tilde{T}}{\partial {\tilde{X}}^{2}}+\frac{\partial^{2}\tilde{T}}{\partial {\tilde{Y}}^{2}}\right).$$

(7) $$\left(\frac{\partial \tilde{F}}{\partial \tilde{t}}+\tilde{U}\frac{\partial \tilde{F}}{\partial \tilde{X}}+\tilde{V}\frac{\partial \tilde{F}}{\partial \tilde{Y}}\right)=D_{b}\left(\frac{\partial^{2}\tilde{F}}{\partial {\tilde{X}}^{2}}+\frac{\partial^{2}\tilde{F}}{\partial {\tilde{Y}}^{2}}\right)+\frac{D_{t}}{T_{0}}\left(\frac{\partial^{2}\tilde{T}}{\partial {\tilde{X}}^{2}}+\frac{\partial^{2}\tilde{T}}{\partial {\tilde{Y}}^{2}}\right).$$

Here,$\;\rho_{e}$ is the electrical charge density, $\rho_{f}$ is the fluid density,$\;\rho_{p}$ is the nanoparticle mass density, $\rho_{0}$ is the nanofluid density at reference temperature $\left(T_{0}\right),\;E_{x}$ is the applied electric field, $\tilde{U}$ represents the velocity along the $\tilde{X}$ direction, $\tilde{V}$ represents the velocity along the $\tilde{Y}$ direction, $\tilde{T}$ is the temperature, $\tilde{C}$ is the solutal concentration, $\tilde{F}$ is the nanoparticle volume fraction, $\tilde{P}$ is the pressure, μ is the fluid viscosity, g is the acceleration due to gravity, $\beta_{t}$ is the volumetric thermal expansion coefficient of fluid, $\beta_{c}$ is the volumetric solutal expansion coefficient of fluid, ${\left(\rho c_{p}\right)}_{f}$ is the heat capacity of fluid, ${\left(\rho c_{p}\right)}_{p}$ is the effective heat capacity of nanoparticle, k is the thermal conductivity, $D_{tc}$ is the Dufour diffusivity, $D_{ct}$ is the Soret diffusivity, $D_{b}$ is Brownian diffusion coefficient, $D_{t}$ is Thermophoretic diffusion coefficient and $D_{s}$ is solutal diffusivity.

The Poisson–Boltzmann equation [26] in a microchannel is defined as

(8) $$\nabla^{2}\tilde{\phi }=-\frac{\rho_{e}}{\in }.$$

Here, $\rho_{e}\left(=-z_{v}e\left({\tilde{n}}^{-}-{\tilde{n}}^{+}\right)\right)$ is the total charge density, ∈ is the dielectric permittivity, $\tilde{\phi }$ is the electric potential, ${\tilde{n}}^{-}$ is the anion, ${\tilde{n}}^{+}$ is the cation and defined as ${\tilde{n}}^{\pm }={\tilde{n}}_{0}e^{\left(\pm \frac{ez_{v}}{T_{av}K_{B}}\tilde{\phi }\right)}$.

Moreover, $n_{0},z_{v},e,K_{B}$ and $T_{av}$ are the bulk concentration, charge balance, electronic charge, Boltzmann constant and average temperature, respectively.

The translational transformation between fixed coordinate system $\left(\tilde{X},\tilde{Y}\right)$ and moving coordinate system $\left(\tilde{x},\tilde{y}\right)$ is [8]

(9) $$\tilde{x}=\tilde{X}-c\tilde{t,}\tilde{y}=\tilde{Y},\tilde{u}\left(\tilde{x},\tilde{y}\right)=\tilde{U}\left(\tilde{X},\tilde{Y},\tilde{t}\right)-c,\tilde{v}\left(\tilde{x},\tilde{y}\right)=\tilde{V}\left(\tilde{X},\tilde{Y},\tilde{t}\right),\;\tilde{p}\left(\tilde{x},\tilde{y}\right)=\tilde{P}\left(\tilde{X},\tilde{Y},\tilde{t}\right),T\left(\tilde{x},\tilde{y}\right)=\tilde{T}\left(\tilde{X},\tilde{Y},\tilde{t}\right).$$

Using the transformations (9) in Equations (2)–(7), after introducing the dimensionless parameters:

(10) $$x=\frac{\tilde{x}}{\lambda },\;y=\frac{\tilde{y}}{\tilde{a}},\;t=\frac{c\tilde{t}}{\lambda },\;u=\frac{\tilde{u}}{c},\;v=\frac{\tilde{v}}{\alpha c},\;p=\frac{{\tilde{a}}^{2}}{\lambda c\mu }\tilde{p},\alpha =\frac{\tilde{a}}{\lambda },\;h=\frac{\tilde{h}}{\tilde{a}},\;n=\frac{\tilde{n}}{n_{0}},m_{e}=\frac{\tilde{a}}{\lambda_{d}},\;\varepsilon =\frac{\tilde{b}}{\tilde{a}},\;\theta =\frac{\tilde{T}-T_{0}}{T_{0}},\Omega =\frac{\tilde{C}-C_{0}}{C_{0}},\gamma =\frac{\tilde{F}-F_{0}}{F_{0}},\lambda_{d}{}^{2}=\frac{\in K_{B}T_{0}}{2n_{0}{\left(ez_{v}\right)}^{2}},\;\phi =\frac{ez_{v}}{T_{av}K_{B}}\tilde{\phi },\;P_{e}=\frac{c\tilde{a}}{D},\;R_{e}=\frac{\rho_{f}c\tilde{a}\;}{\mu },\;\;P_{r}=\frac{\mu {\left(\rho c_{p}\right)}_{f}}{\rho_{f}k},\;U_{hs}=-\frac{T_{av}K_{B}E_{x}\in }{ez_{v}c\mu },\;u=\frac{\partial \psi }{\partial y},\;v=-\frac{\partial \psi }{\partial x},\;G_{rt}=\frac{\rho_{0}g{\tilde{a}}^{2}\beta_{t}T_{0}\left(1-F_{0}\right)}{c\mu },\;f=\frac{q}{c\tilde{a}},\;G_{rc}=\frac{\rho_{0}g{\tilde{a}}^{2}\beta_{c}C_{0}\left(1-F_{0}\right)}{c\mu },\;G_{rf}=\frac{g{\tilde{a}}^{2}\left(\rho_{p}-\rho_{0}\right)F_{0}}{c\mu },\;N_{t}=\frac{D_{t}{\left(\rho c_{p}\right)}_{p}\;}{k},\;S_{s}=\frac{c\tilde{a}\;}{D_{s}},\;S_{b}=\frac{c\tilde{a}\;}{D_{b}},\;N_{b}=\frac{D_{b}{\left(\rho c_{p}\right)}_{p}F_{0}\;}{k},\;N_{ct}=\frac{D_{ct}T_{0}}{D_{s}C_{0}},\;N_{tc}=\frac{D_{tc}C_{0}}{kT_{0}},$$

where $x,y,m_{e},\alpha ,\lambda_{d},p,U_{hs},\varepsilon ,\theta ,\Omega ,\gamma ,P_{e},R_{e},P_{r},G_{rt},G_{rc},G_{rf},N_{t},N_{b},\;N_{ct},\;N_{tc},f$ and Θ are non-dimensional axial coordinate, non-dimensional transverse coordinate, electro-osmotic parameter, peristaltic wave number, Debye length, non-dimensional pressure, Helmholtz-Smoluchowski velocity, amplitude ratio, dimensionless temperature, dimensionless species concentration, dimensionless nanoparticle volume fraction, ionic Peclet number, Reynolds number, Prandtl number, thermal Grashof number, solutal Grashof number, nanoparticle Grashof number, thermophoresis parameter, Brownian motion parameter, Soret parameter, Dufour parameter, dimensionless volume flow rate in moving frame and dimensionless volume flow rate in fixed frame, respectively.

Applying Debye-H$\ddot{u}$ckel linearization approximation [27] and Equation (10), Equation (8) becomes

(11) $$\frac{d^{2}\phi }{dy^{2}}=m_{e}{}^{2}\;\phi ,$$

where $m_{e}$ is the electro-osmotic parameter. The analytical solution of Equation (11) subject to boundary condition $\frac{\partial \phi }{\partial y}=0,\;\;$ at y=0 and ϕ=1, at y=h(x) yeild

(12) $$\phi \left(y\right)=\frac{\cosh\left(m_{e}y\right)}{\cosh\left(m_{e}h\right)}.$$

Using Equation (10), followed by long wavelength approximation [8], Equations (3)–(7) can be reduced to following form:

(13) $$\frac{\partial p}{\partial x}=\frac{\partial^{2}u}{\partial y^{2}}+G_{rt}\theta +G_{rc}\Omega -G_{rf}\gamma +m_{e}{}^{2}U_{hs}\phi ,$$

(14) $$\frac{\partial p}{\partial y}=0,$$

(15) $$\frac{\partial^{2}\theta }{\partial y^{2}}+N_{tc}\frac{\partial^{2}\Omega }{\partial y^{2}}+N_{b}\frac{\partial \theta }{\partial y}\frac{\partial \gamma }{\partial y}+N_{t}{\left(\frac{\partial \theta }{\partial y}\right)}^{2}=0,$$

(16) $$\frac{\partial^{2}\Omega }{\partial y^{2}}+N_{ct}\left(\frac{\partial^{2}\theta }{\partial y^{2}}\right)=0,$$

(17) $$\frac{\partial^{2}\gamma }{\partial y^{2}}+\frac{N_{t}}{N_{b}}\left(\frac{\partial^{2}\theta }{\partial y^{2}}\right)=0.$$

The dimensionless boundary conditions are:

(18a) $$\frac{\partial u}{\partial y}=0,\;\;\theta =0,\;\;\;\;\Omega =0,\;\;\;\;\gamma =0,\;\;\;\mathrm{at}\;y=0,$$

(18b) u=0,    θ=1,  Ω=1,   γ=1, at y=h(x)=1+εsinx,

## 3. Solution Procedure

### Analytical Solution

The analytical solutions [17] of temperature, solutal concentration and nanoparticle volume fraction field are obtained after solving simultaneous Equations (15)–(17) subject to boundary condition (18a,b):

(19) $$\theta =\frac{e^{-N_{0}y}-1}{e^{-N_{0}h}-1},$$

(20) $$\Omega =\frac{N_{t}}{N_{b}}\left(\frac{e^{-N_{0}y}-1}{1-\;e^{-N_{0}h}}\right)+\frac{1}{h}\left(1+\frac{N_{t}}{N_{b}}\right)y,$$

(21) $$\gamma =N_{ct}\left(\frac{e^{-N_{0}y}-1}{1-\;e^{-N_{0}h}}\right)+\frac{1}{h}\left(1+N_{ct}\right)y.$$

Using Equations (19)–(21) in Equation (13) and using boundary condition (18a) and (18b), the analytical solution of axial velocity is obtained as:

(22) $$u=\frac{1}{2}\frac{\partial p}{\partial x}\left(y^{2}-h^{2}\right)-N_{1}\left\{\frac{1}{2}\left(y^{2}-h^{2}\right)-\frac{1}{N_{0}{}^{2}}\left(e^{-N_{0}y}-e^{-N_{0}h}\right)-\frac{1}{N_{0}}\left(y-h\right)\right\}-\frac{N_{2}}{6}\left(y^{3}-h^{3}\right)+\;\;\;\;\;\;\;\;U_{hs}\left(1-\frac{\cosh\left(m_{e}y\right)}{\cosh\left(m_{e}h\right)}\right),$$

where

$$N_{0}=\frac{1}{h}\left(\frac{N_{t}+N_{b}}{1-N_{ct}N_{tc}}\right),\;N_{1}=\frac{1}{1-\;e^{-N_{0}h}}\left(G_{rt}-G_{rc}\Omega N_{ct}+\frac{N_{t}}{N_{b}}G_{rf}\right),\;N_{2}=\frac{1}{h}\left(G_{rc}\left(1+N_{ct}\right)-\left(1+\frac{N_{t}}{N_{b}}\right)G_{rf}\right).$$

The volume flow rate is given by

(23) $$Q=\int_{0}^{h}udy,$$

Using Equation (22) after rearranging the terms, pressure gradient is obtained as

(24) $$\frac{\partial p}{\partial x}=\frac{-3Q}{h^{3}}-N_{1}\left\{\frac{3e^{-N_{0}h}}{N_{0}{}^{3}h^{3}}\left(1+N_{0}h\right)-\frac{3}{N_{0}{}^{3}h^{3}}+\frac{3}{2N_{0}h}-1\right\}+\frac{3N_{2}}{8}h+\frac{3U_{hs}}{h^{3}}\left(h-\frac{\sinh\left(m_{e}h\right)}{m_{e}\cosh\left(m_{e}h\right)}\right).$$

The pressure rise across one wavelength ΔP is as follows:

(25) $$\Delta P=\int_{0}^{1}\frac{\partial p}{\partial x}dx.$$

The volume flow rate in fixed frame is given by

(26) $$Q=\int_{0}^{h}\tilde{U}\left(\tilde{X},\tilde{Y},\tilde{t}\right)d\tilde{Y}.$$

Using Equation (9) in Equation (26) and after integration, we obtain

(27) Q=q+ch.

The time average flow rate over one time period is defined as

(28) $$\tilde{Q}=\int_{0}^{1}Qdt.$$

Using Equations (10) and (27) in Equation (28), we obtain

(29) Θ=f+1.

The stream function $\left(u=\frac{\partial \psi }{\partial y},\;v=-\frac{\partial \psi }{\partial x}\right)$ in wave frame takes the following form:

(30) $$\psi =\frac{1}{2}\frac{\partial p}{\partial x}\left(\frac{1}{3}y^{3}-h^{2}y\right)-N_{1}\left\{\frac{1}{2}\left(\frac{1}{3}y^{3}-h^{2}y\right)+\frac{1}{N_{0}{}^{2}}\left(\frac{1}{N_{0}}e^{-N_{0}y}+ye^{-N_{0}h}+\frac{1}{N_{0}}\right)-\frac{1}{N_{0}}\left(\frac{1}{2}y^{2}-hy\right)\right\}-\frac{N_{2}}{6}\left(\frac{1}{4}y^{4}-yh^{3}\right)-y+U_{hs}\left(y-\frac{\sinh\left(m_{e}h\right)}{m_{e}\cosh\left(m_{e}h\right)}\right).$$

The heat transfer coefficient Z at the wall (y=h(x)) is as follows:

(31) $$Z=h_{x}\frac{\partial \theta }{\partial y}{|}_{y=h}.$$

## 4. Entropy Generation Analysis

The dimensional volumetric entropy generation during nanofluid flow with double diffusive convection is given by

(32) $$S_{gen}=\frac{k}{{\tilde{T}}^{2}}({\left(\frac{\partial \tilde{T}}{\partial \tilde{X}}\right)}^{2}+{\left(\frac{\partial \tilde{T}}{\partial \tilde{Y}}\right)}^{2}))+[\frac{D_{tc}}{\tilde{T}}(\frac{\partial^{2}}{\partial {\tilde{X}}^{2}}+\frac{\partial^{2}}{\partial {\tilde{Y}}^{2}})\tilde{C}+\frac{{\left(\rho C_{p}\right)}_{p}}{\tilde{T}}\{D_{b}(\frac{\partial \tilde{F}\partial \tilde{T}}{\partial \tilde{X}\partial \tilde{X}}+\frac{\partial \tilde{F}\partial \tilde{T}}{\partial \tilde{Y}\partial \tilde{Y}})+\frac{D_{t}}{T_{0}}(({\frac{\partial \tilde{T}}{\partial \tilde{X}})}^{2}+{\left(\frac{\partial \tilde{T}}{\partial \tilde{Y}}\right)}^{2})\}]$$

Equation (32) comprises two parts: one part consists of entropy generation due to heat transfer and the second part consists of entropy generation due to solutal concentration.

The characteristics entropy generation is given as

(33) $$S_{g}=\frac{k}{{\tilde{a}}^{2}}.$$

Using Equations (10) and (33), the non-dimensional volumetric entropy generation can be expressed as

(34) $$N_{s}=\frac{S_{gen}}{S_{g}}=\frac{1}{{\left(1+\theta \right)}^{2}}\frac{\partial^{2}\theta }{\partial y^{2}}+\frac{1}{1+\theta }\left[N_{tc}\frac{\partial^{2}\Omega }{\partial y^{2}}+N_{b}\frac{\partial \theta }{\partial y}\frac{\partial \gamma }{\partial y}+N_{t}{\left(\frac{\partial \theta }{\partial y}\right)}^{2}\right],$$

The total entropy generation or entropy generation number $N_{s}$ in Equation (34) can be written as sum of

(35) $$N_{s}=N_{HT}+N_{DC}.$$

Here, $N_{HT}$ is the dimensionless entropy generation due to heat transfer and $N_{DC}$ represent the dimensionless entropy generation due to solutal concentration.

The thermal irreversibility of the system is defined as the Bejan number $B_{e}$ [38]:

(36) $$B_{e}=\frac{N_{HT}}{N_{HT}+N_{DC}}\;=\;\frac{N_{HT}}{N_{s}}\;=\;\frac{\frac{1}{{\left(1+\theta \right)}^{2}}\frac{\partial^{2}\theta }{\partial y^{2}}}{\frac{1}{{\left(1+\theta \right)}^{2}}\frac{\partial^{2}\theta }{\partial y^{2}}+\frac{1}{1+\theta }\left[N_{tc}\frac{\partial^{2}\Omega }{\partial y^{2}}+N_{b}\frac{\partial \theta }{\partial y}\frac{\partial \gamma }{\partial y}+N_{t}{\left(\frac{\partial \theta }{\partial y}\right)}^{2}\right]}.$$

## 5. Results and Discussion

**Figure 2:**
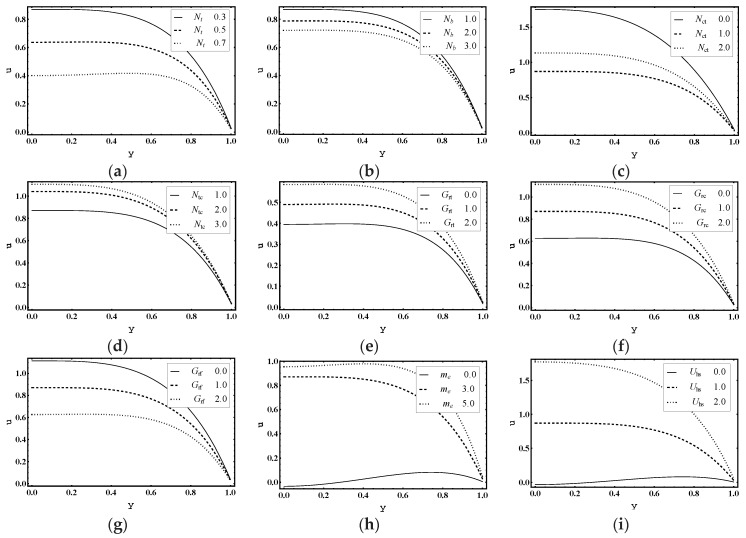
Axial velocity u profile for (**a**) *N_t_*, (**b**) *N_b_*, (**c**) *N_ct_*, (**d**) *N_tc_*, (**e**) *G_rt_*, (**f**) *G_rc_*, (**g**) *G_rf_*, (**h**) *m_e_*, (**i**) *U_hs_*, while other parameters are $\frac{\partial p}{\partial x}=1,x=1,\varepsilon =0.01,\Theta =0.9,\;N_{t}=1,N_{b}=1,N_{ct}=1,N_{tc}=2,G_{rt}=5,G_{rc}=1,G_{rf}=1,\;m_{e}=3,\;U_{hs}=1.$

**Figure 3:**
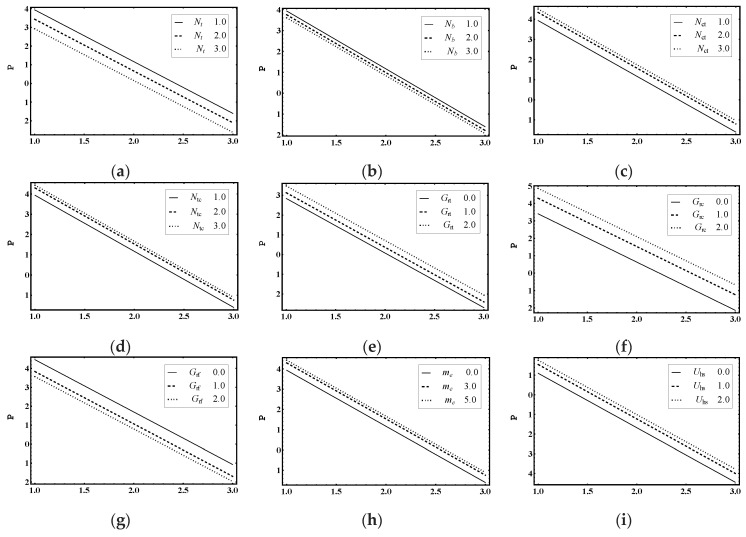
Pressure rise ΔP profile for (**a**) *N_t_*, (**b**) *N_b_*, (**c**) *N_ct_*, (**d**) *N_tc_*, (**e**) *G_rt_*, (**f**) *G_rc_*, (**g**) *G_rf_*, (**h**) *m_e_*, (**i**) *U_hs_*, while the other parameters are $y=0,\varepsilon =0.06,\;N_{t}=1,N_{b}=1,N_{ct}=1,N_{tc}=2,G_{rt}=5,G_{rc}=1,G_{rf}=1,\;m_{e}=3,\;U_{hs}=1.$

**Figure 4:**
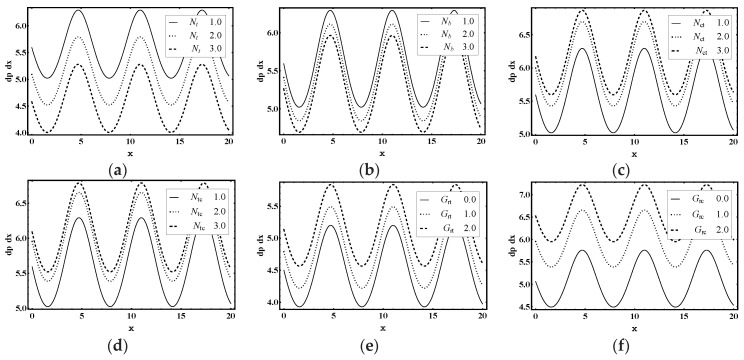

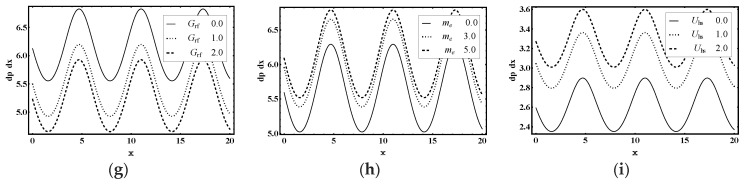
Pressure gradient dp/dx profile for (**a**) *N_t_*, (**b**) *N_b_*, (**c**) *N_ct_*, (**d**) *N_tc_*, (**e**) *G_rt_*, (**f**) *G_rc_*, (**g**) *G_rf_*, (**h**) *m_e_*, (**i**) *U_hs_*, while other parameters are $y=0,\varepsilon =0.06,\;\Theta =0.9,N_{t}=1,N_{b}=1,N_{ct}=1,N_{tc}=2,G_{rt}=5,G_{rc}=1,G_{rf}=1,\;m_{e}=3,\;U_{hs}=1.$

**Figure 5:**
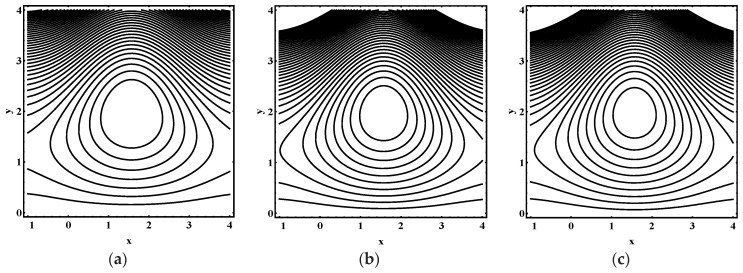
Streamline distribution for (**a**) $N_{t}=1.0$, (**b**) $N_{t}=1.5\;$, (**c**) $N_{t}=2.0$, while other parameters are $\varepsilon =0.06,\;\Theta =0.9,N_{b}=1,N_{ct}=1,N_{tc}=0.1,G_{rt}=0.1,G_{rc}=1,G_{rf}=1,\;m_{e}=0.8,\;U_{hs}=-1.$

**Figure 6:**
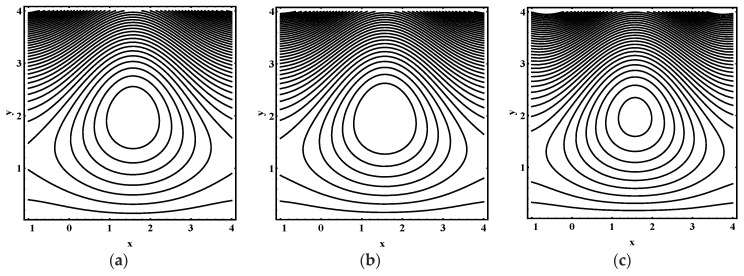
Streamline distribution for (**a**) $N_{t}=1.0$, (**b**) $N_{t}=1.5\;$, (**c**) $N_{t}=2.0$, while other parameters are $\varepsilon =0.06,\;\Theta =0.9,N_{t}=1,N_{ct}=1,N_{tc}=0.1,G_{rt}=0.1,G_{rc}=1,G_{rf}=1,\;m_{e}=0.8,\;U_{hs}=-1.$

**Figure 7:**
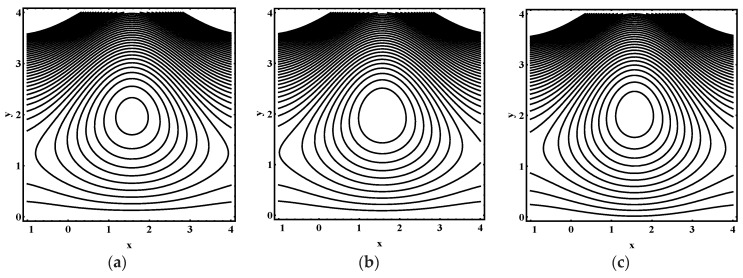
Streamline distribution for (**a**) $G_{rt}=0.0$, (**b**) $G_{rt}=0.1$, (**c**) $G_{rt}=0.2$, while other parameters are $\varepsilon =0.06,\;\Theta =0.9,N_{t}=1,N_{b}=1,N_{ct}=1,N_{tc}=0.1,G_{rc}=1,G_{rf}=1,\;m_{e}=0.8,\;U_{hs}=-1.$

**Figure 8:**
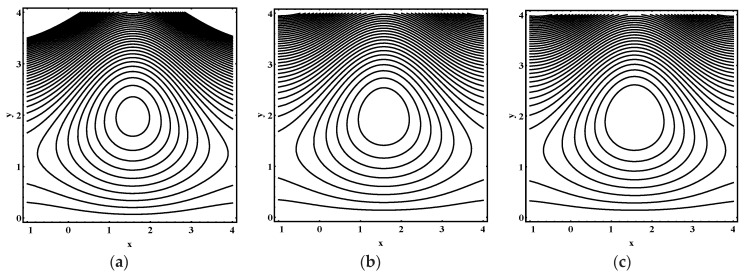
Streamline distribution for (**a**) $\;G_{rc}=1.0$, (**b**) $\;G_{rc}=1.5$, (**c**) $\;G_{rc}=2.0$, while other parameters are $\varepsilon =0.06,\;\Theta =0.9,N_{t}=1,N_{b}=1,N_{ct}=1,N_{tc}=0.1,G_{rt}=0.1,G_{rf}=1,\;m_{e}=0.8,\;U_{hs}=-1.$

**Figure 9:**
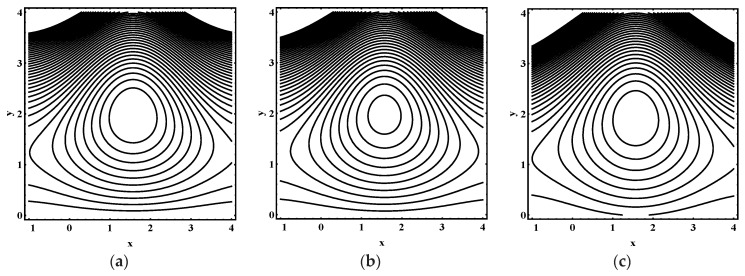
Streamline distribution for (**a**) $G_{rf}=1.0$, (**b**) $G_{rf}=1.5$, (**c**) $G_{rf}=2.0$, while other parameters are $\varepsilon =0.06,\;\Theta =0.9,N_{t}=1,N_{b}=1,N_{ct}=1,N_{tc}=0.1,G_{rt}=0.1,G_{rc}=1,\;m_{e}=0.8,\;U_{hs}=-1.$

**Figure 10:**
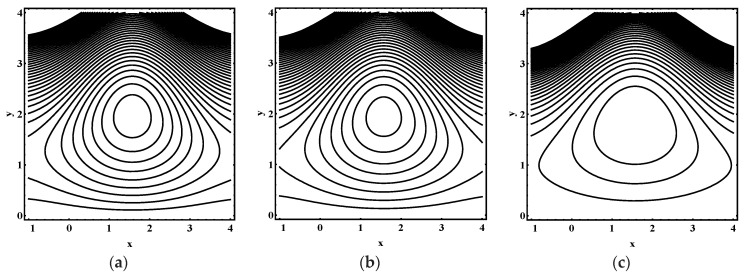
Streamline distribution for (**a**) $m_{e}\to 0.0$, (**b**) $\;m_{e}=1.0$, (**c**) $m_{e}=1.5$, while other parameters are $\varepsilon =0.06,\;\Theta =0.9,N_{t}=1,N_{b}=1,N_{ct}=1,N_{tc}=0.1,G_{rt}=0.1,G_{rc}=1,G_{rf}=1,U_{hs}=-1.$

**Figure 11:**
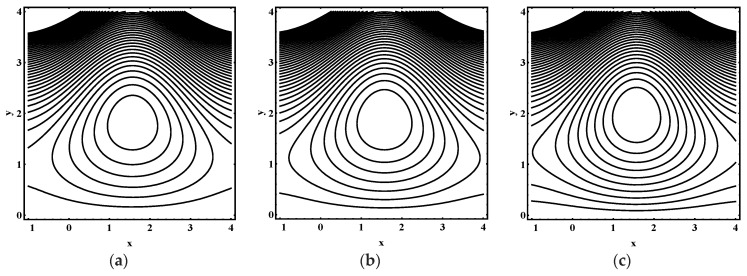
Streamline distribution for (**a**) $U_{hs}=-3.0$, (**b**) $U_{hs}=-2.0$, (**c**) $U_{hs}=1.0$, while other parameters are $\varepsilon =0.06,\;\Theta =0.9,N_{t}=1,N_{b}=1,N_{ct}=1,N_{tc}=0.1,G_{rt}=0.1,G_{rc}=1,G_{rf}=1,\;m_{e}=0.8.$

**Figure 12:**
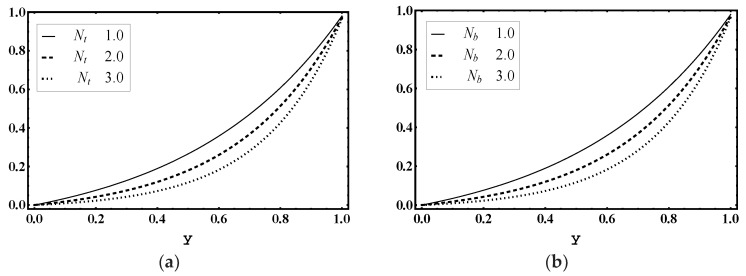

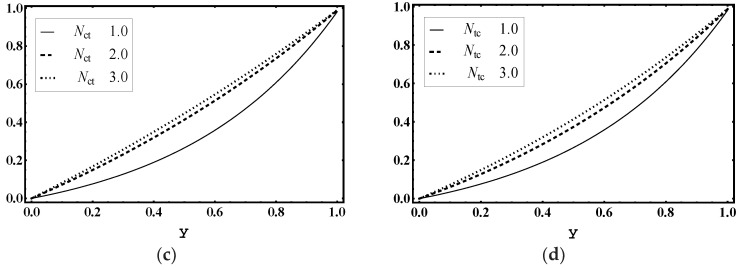
Temperature θ profile for (**a**) *N_t_*, (**b**) *N_b_*, (**c**) *N_ct_*, (**d**) *N_tc_*, while other parameters are $x=1,\varepsilon =0.01,\Theta =0.9,\;N_{t}=1,N_{b}=1,N_{ct}=1,N_{tc}=2,G_{rt}=5,G_{rc}=1,G_{rf}=1,\;m_{e}=3,\;U_{hs}=1.$

**Figure 13:**
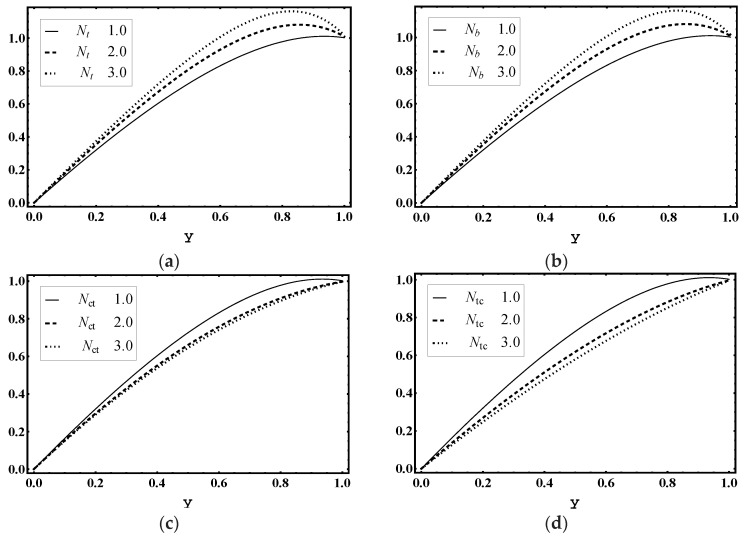
Concentration Ω profile for (**a**) *N_t_*, (**b**) *N_b_*, (**c**) *N_ct_*, (**d**) *N_tc_*, while other parameters are $x=1,\varepsilon =0.01,\Theta =0.9,\;N_{t}=1,N_{b}=1,N_{ct}=1,N_{tc}=2,G_{rt}=5,G_{rc}=1,G_{rf}=1,\;m_{e}=3,\;U_{hs}=1.$

**Figure 14:**
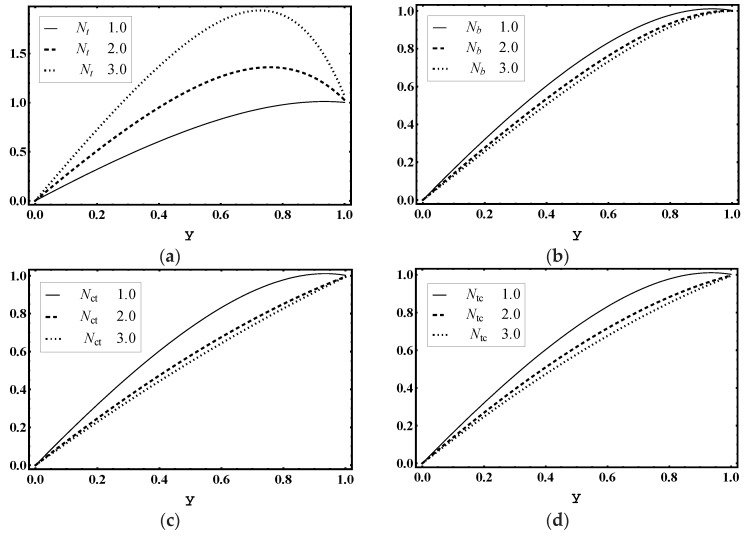
Nanoparticle fraction γ profile for (**a**) *N_t_*, (**b**) *N_b_*, (**c**) *N_ct_*, (**d**) *N_tc_*, while other parameters are $x=1,\varepsilon =0.01,\Theta =0.9,\;N_{t}=1,N_{b}=1,N_{ct}=1,N_{tc}=2,G_{rt}=5,G_{rc}=1,G_{rf}=1,\;m_{e}=3,\;U_{hs}=1.$

**Figure 15:**
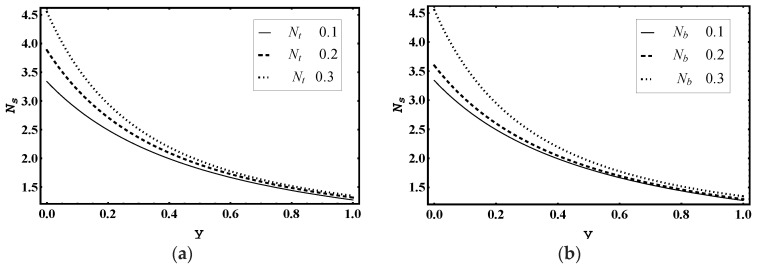

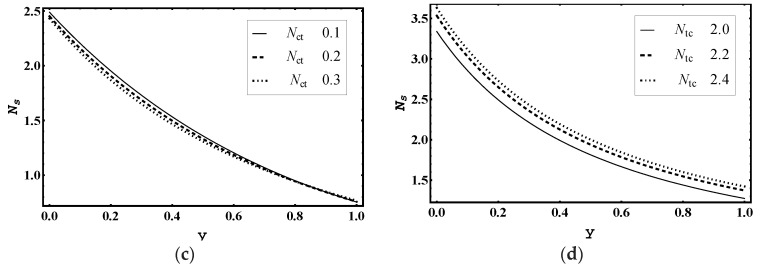
Heat generation number $N_{s}$ profile for (**a**) *N_t_*, (**b**) *N_b_*, (**c**) *N_ct_*, (**d**) *N_tc_*, while other parameters are $x=1,\varepsilon =0.02,\Theta =0.5,\;N_{t}=1,N_{b}=1,N_{ct}=1,N_{tc}=2,G_{rt}=5,G_{rc}=1,G_{rf}=1,\;m_{e}=3,\;U_{hs}=1.$

**Figure 16:**
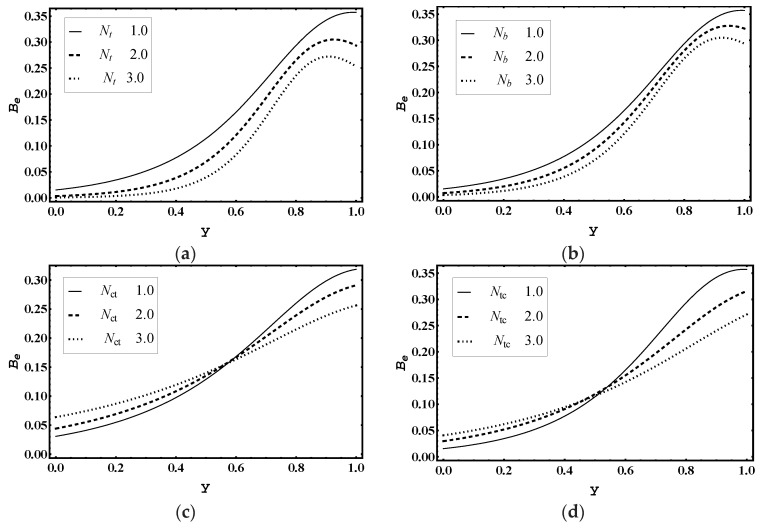
Bejan number $B_{e}$ for (**a**) *N_t_*, (**b**) *N_b_*, (**c**) *N_ct_*, (**d**) *N_tc_*, while other parameters are $x=1,\varepsilon =0.02,\Theta =0.8,\;N_{t}=1,N_{b}=1,N_{ct}=1,N_{tc}=2,G_{rt}=5,G_{rc}=1,G_{rf}=1,\;m_{e}=3,\;U_{hs}=1.$

**Figure 17:**
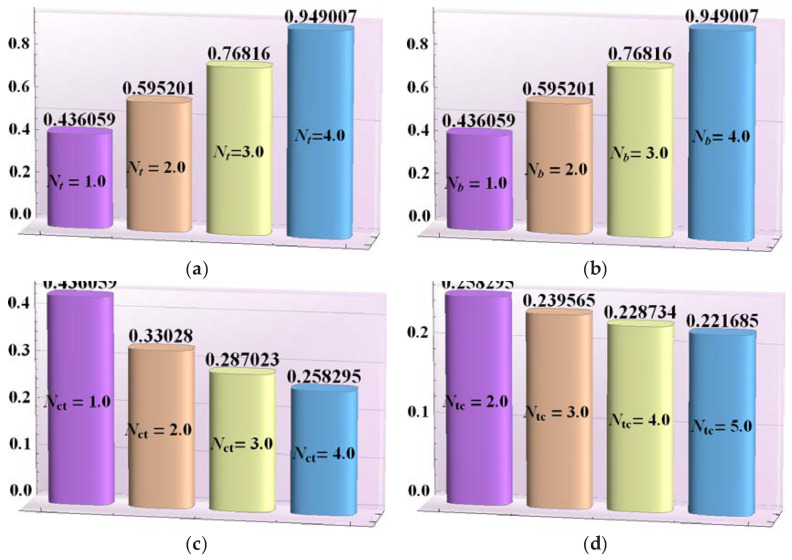
Heat transfer rate Z for (**a**) *N_t_*, (**b**) *N_b_*, (**c**) *N_ct_*, (**d**) *N_tc_*, while other parameters are $x=1,\varepsilon =0.01,\Theta =0.9,\;N_{t}=1,N_{b}=1,N_{ct}=1,N_{tc}=2,G_{rt}=5,G_{rc}=1,G_{rf}=1,\;m_{e}=3,\;U_{hs}=1.$

The main objective of this section is to explain the effects of various parameters (i.e., thermophoresis parameter $N_{t}$, Brownian motion parameter $N_{b}$, Soret parameter $N_{ct}$, Dufour parameter $N_{tc}$, thermal Grashof number $G_{rt}$, solutal Grashof number $G_{rc}$, nanoparticle Grashof number $G_{rf}$, electro-osmotic parameter $m_{e}$ and Helmholtz-Smoluchowski velocity $U_{hs}$) on the velocity profile u, pressure rise ΔP, pressure gradient dp/dx, temperature profile θ, solutal concentration profile Ω, nanoparticle volume faction profile γ_,_ entropy generation number $N_{s}$, Bejan number $B_{e}$ and heat transfer coefficient Z. Moreover, the trapping phenomenon is also illustrated. The graphical results are shown in Figure 2, Figure 3, Figure 4, Figure 5, Figure 6, Figure 7, Figure 8, Figure 9, Figure 10, Figure 11, Figure 12, Figure 13, Figure 14, Figure 15, Figure 16 and Figure 17.

### 5.1. Flow Characteristics

Figure 2a–i is displayed to show the advancements of physical parameters in velocity across the microchannel. Figure 2a,b shows that the magnitude of axial velocity u decreased for increasing values of $N_{t}$ and $N_{b}$. As a result, the deposition of thermophoretic particles was reduced and the central axes of the micro channel had an increased particle accumulation. This sedimentation of the nanoparticles can be adjusted by controlling the thermal gradient, applied externally to the fluid. This is significant as nanoparticle suspension in the nanofluid blocks the fluid flow which affects the system’s performance. Consequently, monitoring the deposition of nanoparticles in the fluid is the main requirement. Figure 2c demonstrates that the magnitude of u was reduced for increasing values of $N_{ct}$, although in Figure 2d, a contrary response is observed for $N_{tc}$. Figure 2e,f portrays that the magnitude of u increased with the increase in $G_{rt}$ and $G_{rc}$. However, it declined for increasing values of $G_{rf}$, as shown in Figure 2g. This demonstrates that the change in concentration of nanoparticles reduced velocity while with a changing temperature and solutal concentration, velocity progresses. Figure 2h predicts that $m_{e}$ will enhance the magnitude of axial velocity u. Since $m_{e}\;$ is the ratio of the channel height to the Debye length $\lambda_{d}$, it indicates that $\lambda_{d}$ is inversely proportional to electric double layer thickness. Hence, more fluid flows in the central region. Figure 2i visualizes that $U_{hs}$ develops the magnitude of u. That means velocity of the fluid can be improved by increasing the magnitude of external electric field.

### 5.2. Pumping Characteristics

Figure 3a–i shows the variation of pressure difference across one wavelength ΔP against the average flow rate Θ. These figures show the linear relationship between ΔP and Θ. The pumping phenomenon was divided into four segments: pumping region (adverse pressure gradient ΔP>0, Positive pumping Θ>0), backward/retrograde pumping (ΔP>0, Θ<0), augmented pumping (favorable pressure gradient ΔP<0, Positive pumping Θ>0) and free pumping (ΔP=0). Figure 3a,b represents ΔP for increasing values of $N_{t}$ and $N_{b}$. This shows that pumping decreased throughout the whole region. This is very helpful in nanofluid drug delivery systems [11]. Figure 3c–i highlights that pumping increased as the Soret parameter $N_{ct}$, the Dufour parameter $N_{tc}$, the thermal Grashof number $G_{rt}$, the solutal Grashof number $G_{rc}$, the nanoparticle Grashof number $G_{rf}$, the electro-osmotic parameter $m_{e}$ and Helmholtz–Smoluchowski velocity $U_{hs}$ increased. Here, ΔP is in a linear relationship with flow rate Θ.

The variation of pressure gradient dp/dx is presented in Figure 4a–i for different physical flow parameters. Figure 4a,b highlights that the magnitude of dp/dx decreased as $N_{t}$ and $N_{b}$ increased. Figure 4c–f shows that the Soret parameter $N_{ct}$, the Dufour parameter $N_{tc}$, the thermal Grashof number $G_{rt}$ and the solutal Grashof number $G_{rc}$ distinctly enhanced the magnitude of dp/dx. Conversely, nanoparticle Grashof number $G_{rf}$ acted to strongly reduce dp/dx values, as shown in Figure 4g. This demonstrates that dp/dx can be controlled by adjusting the $G_{rf}$. However, Figure 4h,i shows that the values of dp/dx were enhanced for increasing values of electro-osmotic parameter $m_{e}$ and the Helmholtz–Smoluchowski velocity $U_{hs}$.

### 5.3. Trapping Characteristics

Trapping for different values of the thermophoresis parameter $N_{t}$, Brownian motion parameter $N_{b}$, Soret parameter $N_{ct}$, Dufour parameter $N_{tc}$, thermal Grashof number $G_{rt}$, solutal Grashof number $G_{rc}$, nanoparticle Grashof number $G_{rf}$, electro-osmotic parameter $m_{e}$ and Helmholtz-Smoluchowski velocity $U_{hs}$ are shown in Figure 5, Figure 6, Figure 7, Figure 8, Figure 9, Figure 10 and Figure 11. Figure 5a–c and Figure 6a–c display streamline structure for different values of $N_{t}$ and $N_{b}$. These reveal that the number and size of trapped bolus increased as $N_{t}$ and *N_b_* increases. Figure 7a–c shows that the size of trapped bolus expanded with increasing $G_{rt}$. However, a reverse trend for solutal Grashof number $G_{rc}$, nanoparticle Grashof number $G_{rf}$ and electro-osmotic parameter $m_{e}$ (shown in Figure 8, Figure 9 and Figure 10). Furthermore, Figure 11a–c discloses that the number of trapped bolus increased for dissimilar values of $U_{hs}$.

### 5.4. Temperature Characteristics

The effects of thermophoresis parameter $N_{t}$, Brownian motion parameter $N_{b}$, Soret parameter $N_{ct}$ and Dufour parameter $N_{tc}$ were examined for heat profile θ. Figure 12a,b demonstrates that the magnitude of temperature distribution θ declines for values of $N_{t}$ and $N_{b}.$ In addition, Figure 12c displays that the magnitude of temperature distribution θ was enhanced for more values of $N_{ct}.$ As, Soret parameter $N_{ct}$ is the ratio of temperature to concentration, hence, bigger $N_{ct}$ stands for a higher temperature. Figure 12d shows that the magnitude of temperature distribution θ was boosted for higher values of $N_{tc}.$ Since $N_{tc}$ shows the contribution of concentration gradient to thermal energy flux in the flow, it is evident that the increase in $N_{tc}$ caused a rise in temperature.

### 5.5. Concentration Characteristics

The influence of thermophoresis parameter $N_{t}$, Brownian motion parameter $N_{b}$, Soret parameter $N_{ct}$ and Dufour parameter $N_{tc}$ are observed for concentration profile Ω. Figure 13a,b concludes that the magnitude of Ω increased for more values of $N_{t}$ and $N_{b}.$ Moreover, Figure 13c displays that the magnitude of Ω decayed for more values of $N_{ct}.$ In addition, Soret parameter $N_{ct}$ is the ratio of temperature to concentration. Hence, higher values of $N_{ct}$ lead to decay in concentration. Figure 13d shows that the magnitude of Ω declined for higher values of $N_{tc}.$

### 5.6. Nanoparticle Volume Fraction Characteristics

The influence of thermophoresis parameter $N_{t}$, Brownian motion parameter $N_{b}$, Soret parameter $N_{ct}$ and Dufour parameter $N_{tc}$ is noticed for nanoparticle volume fraction profile γ. Figure 14a demonstrates that the magnitude of γ increased for more values of $N_{t}$. However, Figure 14b–d shows that the magnitude of γ declined for higher values of Brownian motion parameter $N_{b}$, Soret parameter $N_{ct}$ and Dufour parameter $N_{tc}$.

### 5.7. Entropy Production 

Figure 15a–d illustrates the impact of thermophoresis parameter $N_{t}$, Brownian motion parameter $N_{b}$, Soret parameter $N_{ct}$ and Dufour parameter $N_{tc}$ on entropy generation number $N_{s}$. Figure 15a,b demonstrates that the magnitude of $N_{s}$ increased for added values of $N_{t}$ and $N_{b}.$ This was due to thermal irreversibility, i.e., for higher value of $N_{t}$ and $N_{b},$ thermal irreversibility increased very quickly, near the wall. Moreover, Figure 15c displays that the magnitude of $N_{s}$ decayed for more values of $N_{ct}.$ Figure 15d shows that the magnitude of $N_{s}$ increased for higher values of $N_{tc}.$

Figure 16a–d demonstrates the effect of thermophoresis parameter $N_{t}$, Brownian motion parameter $N_{b}$, Soret parameter $N_{ct}$ and Dufour parameter $N_{tc}$ on Bejan number $B_{e}$. Figure 16a,b implies that the magnitude of $B_{e}$ declined for more values of $N_{t}$ and $N_{b}.$ In addition, Figure 16c displays that the magnitude of $B_{e}$ enhanced for more values of $N_{ct}$ near y=0. Figure 16d shows that the magnitude of $N_{s}$ ascended near y=0 for higher values of $N_{tc}$.

### 5.8. Heat Transfer Coefficient

Figure 17a–d reveals the changes in heat transfer coefficient Z for different values of thermophoresis parameter $N_{t}$, Brownian motion parameter $N_{b}$, Soret parameter $N_{ct}$ and Dufour parameter $N_{tc}$. This infers that for various values of $N_{t}$ and $N_{b}$, the magnitude of heat transfer coefficient Z increased. However, the magnitude of heat transfer coefficient Z declined for bigger values of $N_{ct}$ and $N_{tc}$, respectively.

## 6. Concluding Remarks

A theoretical model for the entropy generation in electro-osmotic peristaltic flow of nanofluid with double-diffusive convection through microchannel is reported. The exact solutions are presented for quantities of interest. The results of this study are similar to the results of [11], when $U_{hs}=0$. Moreover, results of this study are comparable to the results of [6], when $G_{rt}=G_{rc}=G_{rf}=U_{hs}=0$. From the current analysis, we conclude that

- The magnitude of total entropy generation increased as the thermophoresis parameter and Brownian motion parameter increases.
- Soret parameter and Dufour parameter $N_{tc}$ strongly controlld the temperature profile and Bejan number profile.
- The velocity, pressure difference, pressure rise, temperature and Bejan number profile decreased as thermophoresis parameter and Brownian motion parameter increased.
- Electro-osmotic parameter strongly affected the velocity profile.
- The magnitude of pressure difference and pressure gradient enhances in the pumping region with the increase in Soret parameter, Dufour parameter, thermal Grashof number, solutal Grashof number, nanoparticle Grashof number, electro-osmotic parameter and Helmholtz-Smoluchowski velocity.
- The volume and number of trapped bolus increased as the thermophoresis parameter $N_{t}$, Brownian motion parameter, thermal Grashof number and Helmholtz-Smoluchowski velocity increases.
- Heat transfer coefficient, entropy generation number and nanoparticles volume fraction strongly surged with the thermophoresis parameter and the Brownian motion parameter increased.

## Nomenclature

$\tilde{a}$	Half width of channel [L]
$\tilde{b}$	Amplitude of wave [L]
$B_{e}$	Bejan number [-]
c	Wave speed [L/T]
C	Solutal concentration
$C_{0}$	Solutal concentration for lower wall
$c_{f}$	Specific heat $\left[{\mathrm{ML}}^{2}/T^{2}K\right]$
$c_{p}$	Heat capacity of fluid $\left[{\mathrm{ML}}^{2}/T^{2}K\right]$
$D_{b}$	Brownian diffusion coefficient $\left[L^{2}/T\right]$
$D_{t}$	Thermophoretic diffusion coefficient $\left[L^{2}/T\right]$
$D_{s}$	Solutal diffusivity $\left[L^{2}/T\right]$
$D_{ct}$	Soret diffusivity $\left[L^{2}/\mathrm{TK}\right]$
$D_{tc}$	Dufour diffusivity $\left[\mathrm{ML}/T^{3}\right]$
e	Electron charge [C]
$E_{x}$	Electric field $\left[M/T^{3}A\right]$
f	Dimensionless volume flow rate in fixed frame $\left[L^{3}/T\right]$
$\tilde{F}$	Nanoparticle volume fraction
$F_{0}$	Nanoparticle volume fraction for lower wall
g	Acceleration due to gravity $\left[L^{2}/T\right]$
$G_{rt}$	Thermal Grashof number [-]
$G_{rc}$	Solutal Grashof number [-]
$G_{rf}$	Nanoparticle Grashof number [-]
$\tilde{h}$	Transverse vibration of wall [L]
$K_{B}$	Boltzmann constant
k	Thermal conductivity $\left[\;\mathrm{ML}/T^{3}K\right]$
$m_{e}$	Electroosmotic parameter [-]
$\;n^{\pm }$	Positive, negative ions
$n_{0}$	Average number of $n^{+}$ or $n^{-}$ ions
$N_{b}$	Brownian motion parameter [-]
$N_{t}$	Thermophoresis parameter [-]
$N_{ct}$	Soret parameter [-]
$N_{tc}$	Dufour parameter [-]
$N_{s}$	Entropy generation number [-]
$N_{HT}$	Dimensionless entropy generation due to heat transfer [-]
$N_{DC}$	Dimensionless entropy generation due to solutal concentration [-]
$\tilde{P}$	Pressure field [ML/$T^{2}$]
p	Pressure field [-]
$P_{r}$	Prandtl number [-]
$P_{e}$	Peclet number [-]
$R_{e}$	Reynolds number [-]
$S_{c}$	Schmidt number [-]
$\tilde{t}$	Dimensional time [T]
t	Dimensionless time [-]
$\tilde{T}$	Temperature field [K]
$T_{0}$	Temperature of fluid at lower wall [K]
$T_{av}$	Average temperature [K]
$U_{hs}$	Helmholtz-Smoluchowski velocity
$\tilde{U},\tilde{V}$	Dimensional velocity components in stationary frame [L/T]
u,v	Non-dimensional velocity components in wave frame [-]
$\tilde{X},\tilde{Y}$	Dimensional coordinates in stationary frame [L]
x,y	Non-dimensional coordinates in wave frame [-]
$z_{v}$	Valence of ions
Z	Heat transfer coefficient [-]
α	Wave number [-]
$\beta_{t}$	Volumetric thermal expansion coefficient of fluid [1/K]
$\beta_{c}$	Volumetric solutal expansion coefficient of fluid [-]
γ	Dimensionless nanoparticle volume fraction [-]
∈	Dielectric permittivity [-]
ε	Amplitude ratio [-]
Θ	Non-dimensional volume flow rate [-]
θ	Dimensionless temperature [-]
λ	Wavelength of peristaltic wave [L]
$\lambda_{d}$	Debye length [-]
μ	Fluid viscosity [M/LT]
$\rho_{0}$	Nanofluid density at reference temperature $T_{0}$ [M/$L^{3}$]
$\;\rho_{p}$	Nanoparticle mass density [M/$L^{3}$]
$\rho_{f}$	Density of fluid [M/$L^{3}$]
$\rho_{e}$	Net ionic charge density [M/$L^{3}$]
${\left(\rho c\right)}_{f}$	Heat capacity of fluid [${\mathrm{ML}}^{2}$/$T^{2}K$]
${\left(\rho c\right)}_{p}$	Effective heat capacity of nanoparticle [${\mathrm{ML}}^{2}$/$T^{2}K$]
$\tilde{\phi }$	Dimensional electric potential distribution [${\mathrm{ML}}^{2}$/$T^{2}I$]
ϕ	Non-dimensional electric potential distribution [-]
ψ	Dimensional Stream function [$L^{2}$/T]
Ω	Non-dimensional concentration field [-]
